# The SLOE 2.0 becomes the subjective letter of evaluation

**DOI:** 10.1002/aet2.10882

**Published:** 2023-06-22

**Authors:** Andrew Golden, Jennifer Li, Matthew Stull

**Affiliations:** ^1^ Department of Emergency Medicine University Hospitals–Cleveland Medical Center Cleveland Ohio USA; ^2^ Department of Medical Education University of Illinois–Chicago Chicago Illinois USA; ^3^ Case Western Reserve University School of Medicine Cleveland Ohio USA; ^4^ Department of Anesthesia University Hospitals–Cleveland Medical Center Cleveland Ohio USA

The changes to the standardized letter of evaluation (SLOE) in emergency medicine (EM) for the 2022–2023 academic year have concerning implications for our students.^1^ Presented at the Council of Residency Directors in Emergency Medicine (CORD) Academic Assembly in the spring of 2022, the updated SLOE has the potential to place students at a disadvantage by relying on subjective assessments influenced by program‐related factors, rather than exclusively focusing on more objective measures of a student's performance.

The standardized letter of recommendation (SLOR) has been used in EM since it was developed by a CORD task force in 1995.^2^ While it has undergone a number of changes since its introduction including being retitled the SLOE, it has become one of the most important pieces of a medical student's application for EM residency positions.^3^, ^4^, ^5^

The final “global assessment” section of the previous SLOE included two questions that were particularly meaningful for SLOE reviewers. Question 1 read, “Compared to other EM residency candidates you have recommended in the last academic year, this candidate is in the:” with the writer to select top 10%, top 1/3, middle 1/3, or lower 1/3. Question 2b read, “How highly would you estimate the candidate will reside on your rank list?” with the same response options as above. Question 1 (hereinafter referred to as “objective global assessment”) allowed for a more objective assessment of a student's performance, while the response to Question 2b (hereinafter referred to as “subjective program ranking”) has the potential to be influenced by a student's specific fit with a residency program's values, culture, or mission.

For the 2022–2023 academic year, the SLOE has undergone revisions that warrant serious consideration and discussion.^1^ The updated SLOE removed the objective global assessment while keeping the subjective program ranking and introducing a new question about the anticipated guidance a student may need. These changes are in contrast to suggestions from prior research indicating the objective global assessment item had a greater number of program directors rating it as being a “good question” when compared to the subjective program ranking item.^3^ Additionally, almost 25% of program directors indicated the subjective program ranking question “needs work” or “should be dropped” compared to only 10% for the objective global assessment.^3^

The change to the updated SLOE creates a number of challenges for students, SLOE writers, and SLOE reviewers. Primarily, despite a call for the initial iteration of the SLOR to provide “objective, comparative, and narrative information,” the newest version of the SLOE places greater reliance on a question that inherently is more subjective and prone to influence by factors external to the student.^2^ Furthermore, previous studies have demonstrated divergent ratings in the objective global assessments and subjective program ranking items for 19% of EM applicants, 68% of whom were scored lower on the subjective program ranking component.^6^ Relying on the subjective program ranking item as a marker of EM performance, as many programs do, may place a large number of students at an unfair disadvantage.

Consider a student who garnered outstanding workplace‐based assessments and scored highly on their end‐of‐clerkship assessment during their EM away rotation but is interested in a fellowship opportunity or serving a patient population unavailable at the site of their rotation. Alternatively, consider a student who performed well clinically on their EM away rotation but did not fit with a specific program's culture. Based on the revised SLOE, both of these students may be assessed lower on the subjective program ranking question despite having excellent potential as EM physicians. This change handicaps students. In circumstances such as these, SLOE writers, now more than ever, must provide more explicit narratives characterizing institution‐specific reasons why students may be assessed lower on this question. Similarly, selection committees must be willing to look past “lower 1/3” or “middle 1/3” assessments on the subjective program ranking item when narrative data suggests a student has potential to thrive within a specific type of training program or environment.

We urge for substantive research to measure the influence the updated SLOE has had throughout this academic year on residency recruitment and selection. Specifically, investigation into how the new questions within the final section of the SLOE are perceived and utilized by SLOE writers and residency selection committees should be prioritized. While the newly integrated question about anticipated support and guidance may help fill the gap of the objective global assessment, research is needed to inform our interpretation of this data. Additionally, further research into discrepancies between narrative data and the subjective program ranking question on the revised SLOE is warranted.

If, as a community, we continue with norm‐referenced assessment in the SLOE, we advocate reinstating the objective global assessment on future iterations as a more objective marker of a student's performance during their EM rotation. This fundamental change recaptures the essence of the SLOE, allowing programs to decide for themselves how a student will end up on their own rank list.
